# Classification-driven framework to predict maize hybrid field performance from metabolic profiles of young parental roots

**DOI:** 10.1371/journal.pone.0196038

**Published:** 2018-04-26

**Authors:** Francisco de Abreu e Lima, Lothar Willmitzer, Zoran Nikoloski

**Affiliations:** 1 Max-Planck Institute of Molecular Plant Physiology, Potsdam, Germany; 2 Bioinformatics Group, Institute of Biochemistry and Biology, University of Potsdam, Potsdam, Germany; Austrian Federal Research Centre for Forests BFW, AUSTRIA

## Abstract

Maize (*Zea mays* L.) is a staple food whose production relies on seed stocks that largely comprise hybrid varieties. Therefore, knowledge about the molecular determinants of hybrid performance (HP) in the field can be used to devise better performing hybrids to address the demands for sustainable increase in yield. Here, we propose and test a classification-driven framework that uses metabolic profiles from *in vitro* grown young roots of parental lines from the Dent × Flint maize heterotic pattern to predict field HP. We identify parental analytes that best predict the metabolic inheritance patterns in 328 hybrids. We then demonstrate that these analytes are also predictive of field HP (0.64 ≥ *r* ≥ 0.79) and discriminate hybrids of good performance (accuracy of 87.50%). Therefore, our approach provides a cost-effective solution for hybrid selection programs.

## Introduction

Hybrid breeding is a key contributor to yield gain in maize (*Zea mays* L.) and offers the means to meet the demands of the growing population [1–5]. Genetic markers have been widely used to predict hybrid performance in different crop species [6–9] with moderate accuracies that vary with respect to the trait of interest (e.g. *r* of 0.74–0.75 for grain yield and 0.88–0.99 for grain dry matter content in maize [10]). This approach can explain the genetic contribution and neglects the often significant environmental effect on hybrid performance with respect to different traits. Another strategy to improve hybrid breeding is to determine the extent to which field hybrid performance (HP) can be predicted from few quantitative molecular (e.g. metabolic) profiles of *in vitro* grown parental lines. Such an approach provides a cost-effective solution that has the potential to bridge the gap between greenhouse and field. Yet, most existing approaches of this kind either have been tested on small designs [11, 12] or use molecular profiles from the same conditions in which HP is assessed [13, 14].

Understanding the relation between HP and molecular profiles of parents has the potential to identify the determinants of specific heterotic traits, such as those relating to yield. As a result, modern omics technologies (e.g. transcriptomics, proteomics, and metabolomics) have emerged as competitive alternatives to classic genetic markers to predict HP in maize [10, 15–17]. Metabolomics provides quantitative data about small molecules whose pools are jointly affected by the genotype and environment [18]. Metabolic profiles of hybrids have already been used to predict HP in maize [14, 19] and rice [20, 21]. The modeling evidence from a small diallel design suggests that the distance of the metabolic profiles of the hybrids from the average metabolic profile over the best performing hybrids correlated negatively with root biomass [11]. Refining this idea on a broader maize Dent × Flint panel, it was shown that the metabolites with particularly robust profiles over the population of hybrids are predictive of field HP [19]. However, the selection based on such modeling strategy requires tremendous resources, since hybrids need to be created, grown, and molecularly assessed. The ultimate prediction scenario is to use few metabolic profiles gathered from early developmental stages of parental lines, preferably grown in cost-effective designs, to predict HP in the field.

Here, we validate and expand the applicability of a classification-driven modeling framework [12] by using metabolic profiles from young roots of 328 test-crosses of the Dent × Flint heterotic pattern as well as the corresponding parental genotypes. The framework is based on the idea that parental metabolites predictive of metabolic inheritance patterns (mIPs) are also predictive of HP (S1 Fig). We demonstrate that parental analytes predictive of mIPs in young roots are predictive of hybrid biomass in the field, thus offering a new application of the classification-driven framework to significantly improve current hybrid breeding programs.

## Results

**Fig 1:**
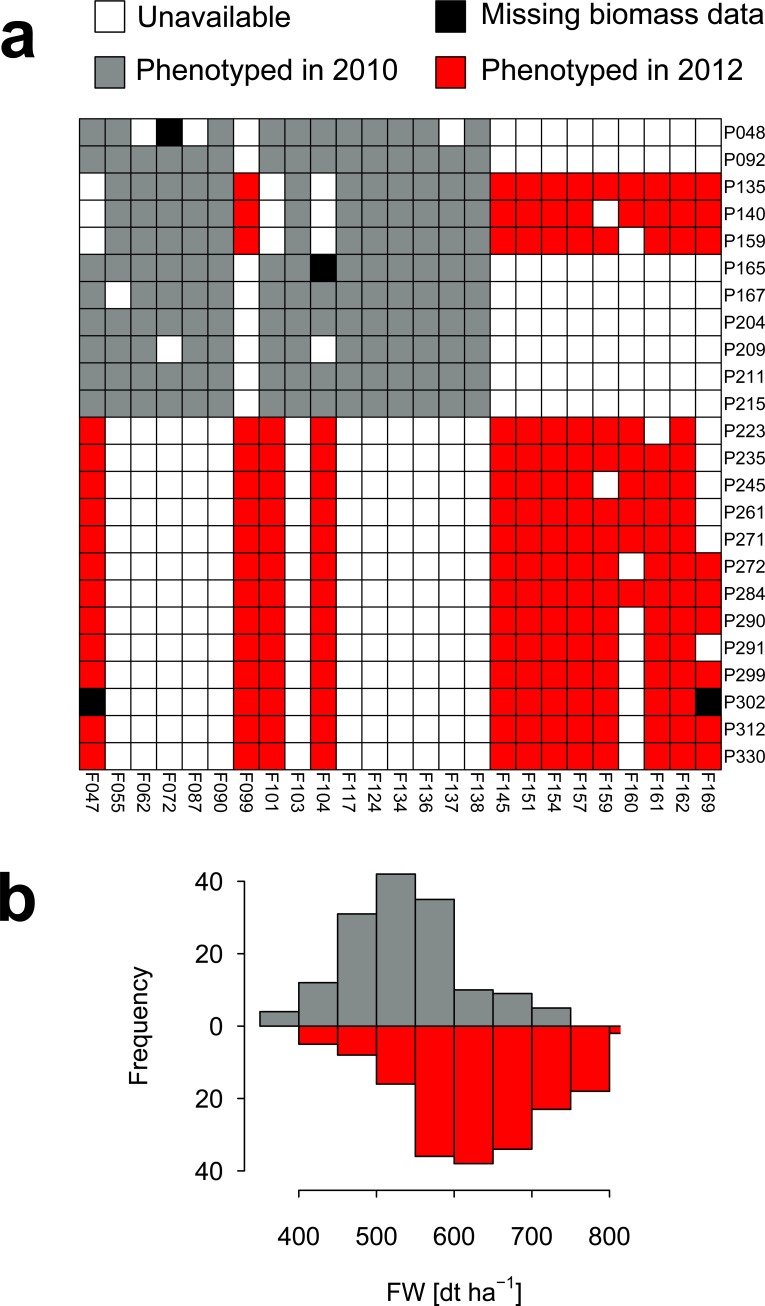
Schematic representation of the Dent × Flint partial factorial mating design and distribution of field biomass. (**a**) Cells denote the crosses between the 24 Dents (rows) and the 25 Flints (columns). Filled cells represent profiled hybrids, with black denoting those with missing phenotyping data. Grey and red cells represent profiled hybrids that were phenotyped in the trials of 2010 and 2012, respectively. (**b**) Distribution of field biomass in hybrids evaluated in 2010 (grey, upper histogram) and 2012 (red, lower histogram).

Our study is based on data from a previously reported partial factorial breeding panel of the European maize Dent × Flint heterotic pattern [19]. This panel comprises 24 Dent lines, 25 Flint lines and 332 Dent × Flint hybrids from crosses thereof. The metabolic profiles for 269 analytes were obtained from the roots of 3.5-day old seedlings. To remove redundancy, we retained only those analytes that were, on average, less correlated to the rest (cf. Methods), resulting in 136 analytes of which 106 were annotated metabolites. From the 332 evaluated hybrids, the performance (i.e., biomass in the field) was assessed in two different field trials for 328, with 148 in 2010 and the remaining 180 in 2012 (Fig 1A, S1 Dataset, cf. Methods). The hybrids evaluated in 2012 exhibited, on average, greater performance compared to the hybrids evaluated in 2010 (two-tailed *t*-test *P* = 2.89 × 10^−23^, Fig 1B).

### Encoding of the hybrid analytes using the metabolic inheritance patterns (mIPs)

**Fig 2:**
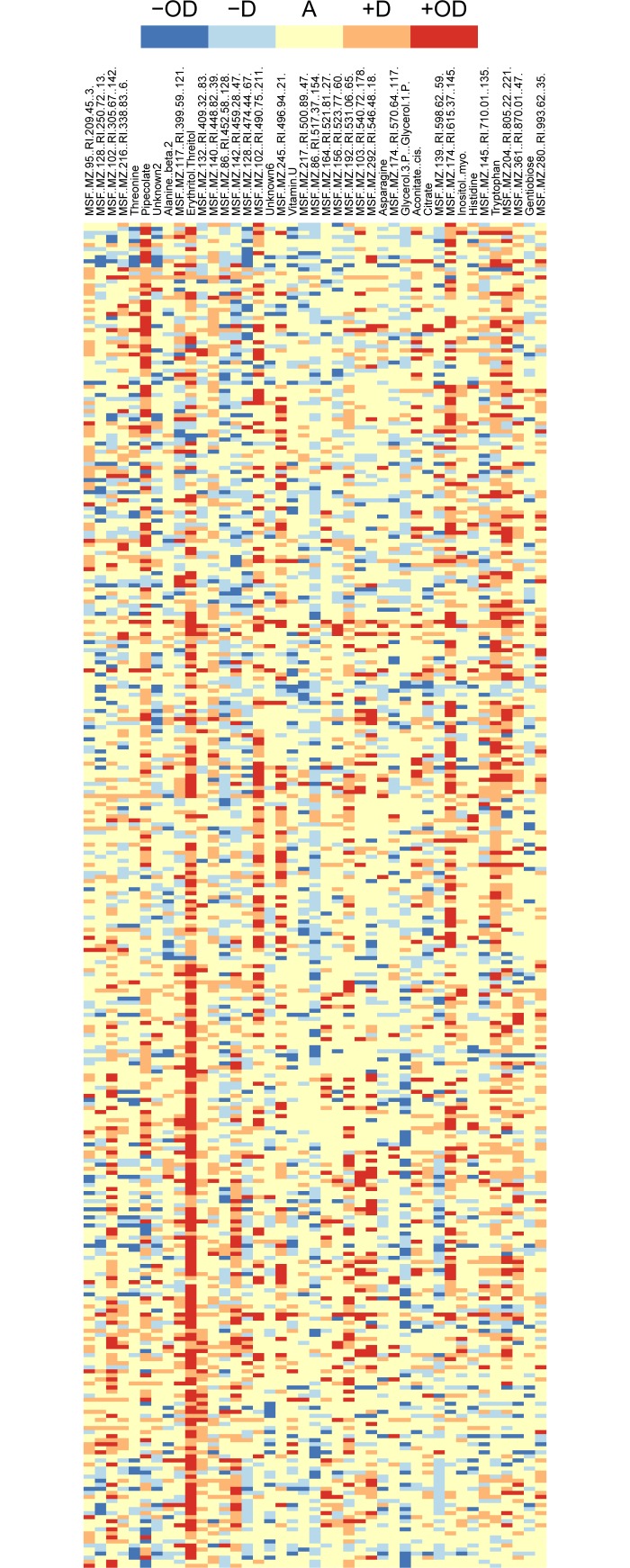
Class distribution of analytes used in the classification-driven framework. Columns correspond to the selected 41 analytes, whereas rows correspond to the 332 crosses. Red, orange, yellow, blue and navy blue colors represent positive overdominance, positive dominance, additivity, negative dominance and negative overdominance, respectively.

First, we assigned class labels for every analyte in each hybrid by classifying the mIP based on the concepts of additivity, dominance and overdominance [12] (S1C Fig). We applied moderated *t*-tests to determine the significance and sign of the differences in the levels of each of the 136 analytes, which we then used to assign labels to the analytes in every hybrid. To this end, we employed the following procedure: If the hybrid level was (*i*) significantly greater/smaller than both parental levels (positive/negative overdominance), mIP was set to +/-2, respectively; (*ii*) significantly greater/smaller than a single parent (positive/negative dominance), mIP was +/-1, respectively; (*iii*) indistinguishable from both parents or greater than one and smaller than the other (additivity), mIP was 0. The resulting matrix of class labels indicated clear overrepresentation of additivity over all crosses and analytes (S1 Table). To avoid excessive class unbalance, we removed analytes with over- or under-representation of the five classes (cf. Methods). As a result, 41 encoded analytes were kept for further analysis, each with labels for the 332 hybrids (Fig 2).

### Classification models predictive of mIPs

**Fig 3:**
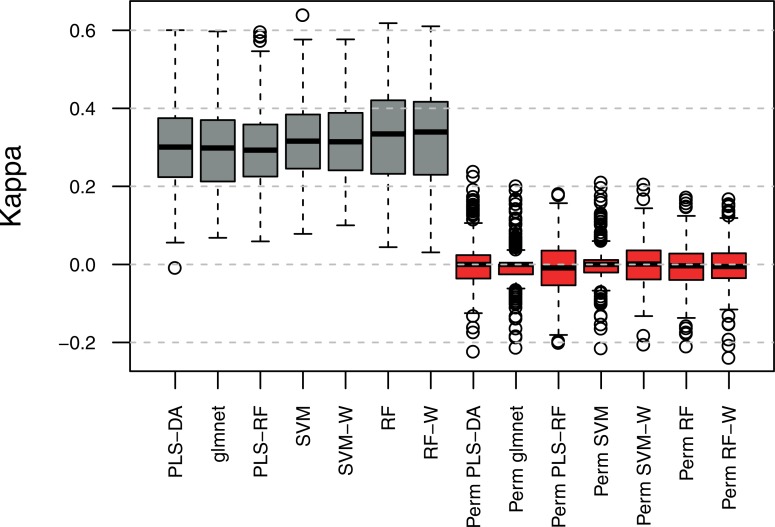
Comparison of classifiers’ performance. Each boxplot summarizes Kappa for classifying the metabolic inheritance patterns (mIPs) from 41 encoded analytes, each in five independent repetitions, with the following methods: linear discriminant analysis following a dimension-reducing partial least-squares transformation (PLS-DA, CV = 0.372), logistic regression with the elastic net (glmnet, CV = 0.370), random forests following a dimension-reducing partial least-squares transformation (PLS-RF, CV = 0.347), support vector machines (SVM, CV = 0.331), support vector machines with class-specific weights (SVM-W, CV = 0.321), random forests (RF, CV = 0.371) and random forests with class-specific weights (RF-W, CV = 0.374). Grey and red colors denote Kappa for the observed and permuted mIPs, respectively.

We then constructed an *n*_crosses_ × (2*n*_analytes_) matrix of predictors, in which each row corresponds to a hybrid and the columns to the analytes in the Dent concatenated to those from the Flint parents. Since the levels of metabolites are mutually dependent due to the underlying metabolic and regulatory networks, we used all 2*n*_analytes_ = 272 analytes to predict the mIPs of the 41 selected. To this end, we compared the performance of seven classification methods: linear discriminant analysis following a dimension-reducing partial least-squares transformation (PLS-DA) [22], logistic regression with the elastic net (glmnet) [23] random forests following a dimension-reducing partial least-squares transformation (PLS-RF) [24, 25], support vector machines (SVM) [26], support vector machines with class-specific weights (SVM-W), random forests (RF) [24] and random forests with class-specific weights (RF-W). The models were built to optimize a measure called Kappa that allows for comparison of classifiers; it quantifies how closely the results of the classification match the ground truth, while controlling for the accuracy of a random classifier as measured by the expected accuracy. In addition, we considered class-specific weights to increase the importance of correctly classifying non-additive mIPs (cf. Methods). We did not observe large differences in the distributions of Kappa values over the seven classifiers for the 41 investigated analytes, each modeled in five independent repetitions. The smallest median Kappa value 0.293 (PLS-RF) suggests moderate but significant performance, since the classifiers for permuted mIPs exhibited the largest average Kappa of only 0.001 (SVM-W, Fig 3). While there were two analytes for which the average Kappa over the seven classifiers was smaller than 0.20, the mIPs of 10 analytes (e.g. glycerol-3-phosphate, erythritol-threitol and gentiobiose) were consistently predicted with Kappa greater than 0.35 (S2 Table). A classifier with small variance and a large mean of Kappa over the 41 investigated analytes provides a reliable performance. Therefore, we compared the classifiers with respect to the coefficients of variation (CV = standard deviation / mean) and focused on SVM-W, which exhibited the smallest CV (0.321) over the analyzed models for ranking the predictors.

### Ranking of the parental analytes and prediction of HP

**Fig 4:**
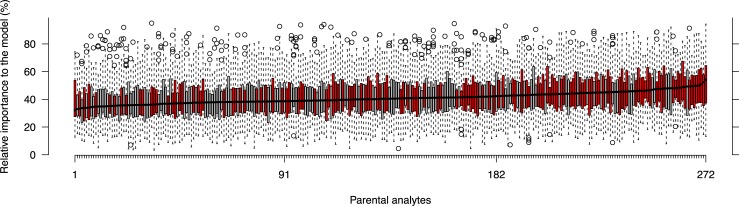
Ranking of parental features based on the decreasing median relative importance to the SVM-W classifier. The median relative importance to the model (%) of the various Dent (maternal, red) and Flint (paternal, grey) analytes exhibits an approximately linear decrease along the ranking (right to left).

**Fig 5:**
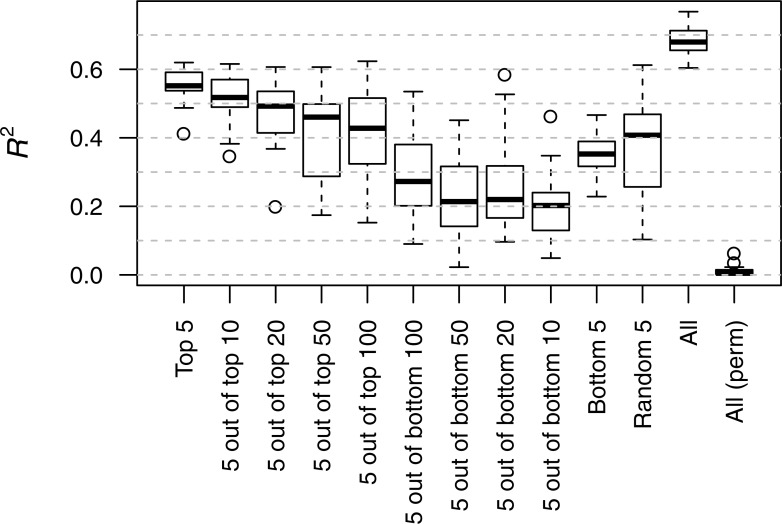
Predictability of hybrid performance based on subsets of the ranked parental features. Hybrid field performance was predicted using support vector regression models (SVR), each trained with different subsets of parental features, based on the median relative importance for predicting the metabolic inheritance patterns (mIPs). The models were trained with the top five parental features (‘Top 5’); five randomly drawn from the top 10/20/50/100 features (‘5 out of top 10’/‘5 out of top 20’/‘5 out of top 50’/‘5 out of top 100’, respectively); five randomly drawn from the bottom 10/20/50/100 features (‘5 out of bottom 10’/‘5 out of bottom 20’/‘5 out of bottom 50’/‘5 out of bottom 100’, respectively); bottom five parental features (‘Bottom 5’); five randomly drawn from all features (‘Random 5’). As the subsets comprise analytes ranked lower, predictability (*R*^2^) decreases. The full model (‘All’) exhibits the highest median *R*^2^ whereas upon permutation of the values of hybrid performance (‘All (perm)’) the median *R*^2^ is almost null.

To rank a parental analyte, we used its relative importance in predicting the mIP across all 41 investigated analytes (cf. Methods). The median importance ranged from 32.16% and 54.03% and its standard deviation from 9.55% and 20.27% (Fig 4). Furthermore, we found no association between the order of both paternal and maternal ranks, as indicated by a Kendall’s *τ* of 0.03 (*P* = 0.58). We then tested if parental analytes predictive of mIP are also predictive of HP in the field. For this purpose, we predicted hybrid performance based on support vector regression models (SVR) [27], each trained with different subsets of parental analytes, based on the previously computed ranking. For this purpose, we trained SVR in twelve scenarios with usage of different number of analytes ranked at the top, bottom, or drawn randomly from the ranking (cf. Methods). We found that predictability (accessed by the coefficient of determination, *R*^2^) from models trained with the five top ranked parental analytes (median *R*^2^ = 0.552) was significantly higher than that from models trained with the five bottom ranked parental features (median *R*^2^ = 0.353, two-tailed *t*-test *P* = 1.70 × 10^−17^). In addition, models trained with analytes positioned lower in the ranking tended to show, on average, lower predictabilities. Furthermore, and expectedly, optimal test prediction was achieved when all analytes were employed (median *R*^2^ = 0.679). The permutation of the biomass data, in turn, led to comparatively poor predictability (median *R*^2^ = 0.005), demonstrating that parental analytes are predictive of HP in the field (Fig 5).

### Classification models for qualitative HP

**Fig 6:**
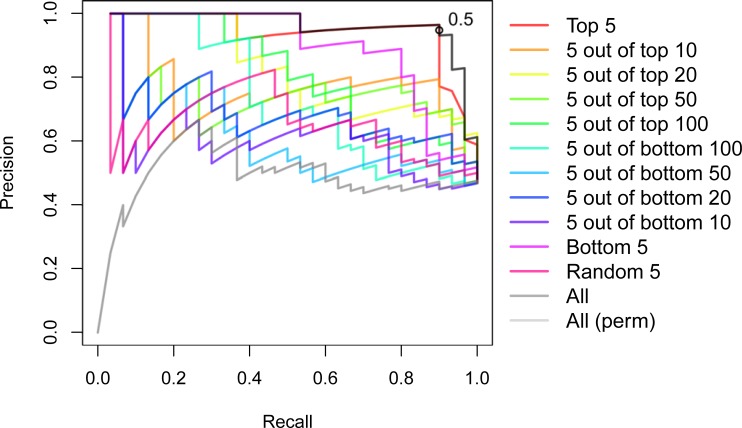
Precision-recall curves from predicting ‘good’ and ‘bad’ performers based on subsets of the ranked parental features. ‘Bad’ and ‘good’ performers were predicted using support vector machines (SVM), each trained with different subsets of parental features, based on the median relative importance for predicting the heterotic mode of action. The models were trained with the top five parental features (‘Top 5’); five randomly drawn from the top 10/20/50/100 features (‘5 out of top 10’/‘5 out of top 20’/‘5 out of top 50’/‘5 out of top 100’, respectively); five randomly drawn from the bottom 10/20/50/100 features (‘5 out of bottom 10’/‘5 out of bottom 20’/‘5 out of bottom 50’/‘5 out of bottom 100’, respectively); bottom five parental features (‘Bottom 5’); five randomly drawn from all features (‘Random 5’). In each model, the class probabilities attributed to the hybrids in the test set were then used to compute precision and recall over multiple probability cutoffs ranging from 0 to 1. The open circle locates the point in the curve of ‘Top 5’ in which the probability cutoff is 0.5.

From a breeder’s perspective, distinguishing between hybrids of good and bad performance is more efficient and profitable than quantitative predictions of HP. Here a hybrid is considered of ‘good’ performance if its HP in the field was above a given threshold; all other hybrids were referred to as ‘bad’. We then built classification models with SVM and different thresholds for labeling the hybrids as ‘good’ or ‘bad’. Our findings demonstrated that the test accuracy was consistent with the predictabilities obtained in the regression setting. The highest median test accuracy (87.50%, with a corresponding Kappa value of 0.748) was obtained from models trained with the five top ranked parental features and the average used as a threshold (S2 and S3 Figs). Similar findings were obtained when the resulting models were assessed based on the area under the receiver operating characteristic curve (AUC). The AUC from the model trained with the top five parental features (median AUC = 0.875) was slightly smaller compared to the AUC from the full model (median AUC = 0.914, S4 Fig). An efficient hybrid selection program must aim at high precision, which amounts to minimizing the number of false positives. However, since there is a trade-off between precision and recall, we also compared the models with respect to the corresponding precision-recall curves. An optimal compromise between the precision and recall was observed for the models having all parental features and the five top ranked parental features. Moreover, we noted that for the latter, the probability cutoff of 0.5 (the default in two-class problems) provides a good compromise between the two measures, with precision and recall taking the values of 0.964 and 0.900, respectively (Fig 6).

## Discussion

We refined and substantially expanded a two-step classification-driven framework that enables us to predict quantitative and qualitative HP performance in the field from the metabolic profiles of parental lines in early developmental stages, grown under *in vitro* conditions. Accurate prediction of HP from molecular markers has the potential to ultimately pinpoint the determinants of heterosis. Heterotic performance has long been regarded as mainly driven by nonadditive genetic variance, a paradigm that motivated existing hypotheses such as dominance, overdominance, pseudooverdominance and epistasis [28]. However, in our large design and across the set of modeled analytes, we observed no correlation between the proportion of analytes exhibiting non-additive mIPs and hybrid performance [29] (S1 Note). Instead, we demonstrated that the metabolic inheritance pattern (mIP) in each of the selected analytes in hybrids can be effectively predicted from the levels of analytes measured in the corresponding parents, as confirmed by permutation of labels. We assessed the classification performance based on Kappa, as a robust metric that contrasts the observed accuracy against the proportion of any potentially overrepresented class [30]. Therefore, Kappa is more penalized in models that misclassify dominance and overdominance. Finally, some models were also equipped with the so-called class-specific weights, which directly define the relative cost of misclassifying particular classes. In our setting, we defined a range of values that increased from additivity to dominance to overdominance, to attribute greater importance to the correct classification of the least representative classes. Interestingly, all seven classifiers tested exhibited similar average performances.

For ranking parental analytes we selected the method having the smallest coefficient of variation with respect to Kappa, i.e. the support vector machine with class-specific weights (SVM-W). Minimization of the coefficient of variation favors the classifiers with high average but also low variance, which is highly relevant in our setting with multiple responses. Based on the cumulative relative frequencies of the Flint and Dent analytes along the ranking, our results suggested that maternal (Dent) analytes were more important in the prediction of mIP compared to the paternal (Flint) analytes [7] (S5 Fig). In addition, we found no association between the orders of the two separate parental analyte ranks, suggesting maternal (Dent) and paternal (Flint) analytes exhibit different predictabilities of mIP. The early metabolism and vigor of maize seedlings is highly dependent on the endosperm composition and quality. The endosperm of maize is a triploid tissue that results from the combination of the paternal sperm with the two maternal polar nuclei in the embryo sac. Therefore, not only are extra-nuclear factors maternally inherited (i.e. mitochondria and plastids), but also the nuclear genetic dosage from the maternal parent (2/3) is twice that from the paternal parent (1/3) [31, 32]. As a result, this skewness in the genetic contribution should be reflected at the metabolic level, as seen in our setting, thus offering a putative explanation why maternal (Dent) analytes act as better predictors of mIP and consequently of HP. However, here we do not consider reciprocal hybrids and consequently cannot assert whether this is the result of parent-of-origin effects.

Finally, we found that parental analytes predictive of mIP were also predictive of HP in both quantitative and qualitative manners. In attempt to dissect the relevance of the rank to the predictability (i.e. *R*^2^) of HP, we compared models trained with different subsets of parental analytes based on their indices along the rank. As the subset comprised parental analytes ranked higher, the greater the predictability of the corresponding model, up to a point in which the difference in predictability between the best five features (*R*^2^ = 0.554) and the full model (*R*^2^ = 0.682) is negligible compared to the difference in the number of features used in each one (5 and 272, respectively). Interestingly, all the best five features are Dent (maternal) analytes, rendering the prediction of HP in two distinct field trials possible without Flint (paternal) data. These five features included threonine, xylose and valine.

The usage of a maximum mIP class balance threshold aimed at filtering out encoded analytes that *i*) display too few instances of underrepresented classes to cover the folds employed in the cross-validation procedure, and *ii*) yield poorly performing models, consequently biasing the ranking of the parental analytes. When we alternatively set a threshold of 0.9 (as opposed to 0.75), we obtain a total of 74 encoded analytes (as opposed to 41), which upon training for analyte ranking ultimately disrupt the predictive ability of the top ranked analytes. More specifically, the prediction from all different subsets of analytes at the threshold of 0.9 is as accurate as using a random subset of analytes of the same size (S6 Fig).

While the earlier study employed a small design with only 12 hybrids, seedling primary root biomass and targeted metabolomics analysis [12], here we analysed 332 hybrids, whole-plant fresh weight per unit of area (dt ha^-1^) determined from two separate field trials and untargeted (thus more comprehensive) metabolomics analysis. In terms of predictability, the correlation of observed and predicted HP using the five best features reported by Feher and co-workers ranged from 0.20 to 0.80 [12]. In our broader panel, the correlation ranged from 0.64 to 0.79 ($r=\sqrt{R^{2}}$), a comparatively narrower interval likely stemming from our larger sample size. Furthermore, the previously reported genomic prediction of HP (i.e. dry weight) with the same population used here resulted in correlations ranging from 0.25 to 0.90 [17], highlighting the comparative performance of our approach in comparison to the contenders. In addition, we expanded the approach to demonstrate that the two-step classification-driven framework can effectively distinguish between ‘good’ and ‘bad’ performing hybrids (accuracy of 87.50%).

One of the pressing points in prediction of hybrid performance with respect to a given traits is the contribution of the potential genotype by environment (GxE) interaction component of the trait’s variability. The gold standard approaches for HP prediction based on genomic markers can explain the genotype proportion of the variance and would be suitable for traits with large heritability. On the other hand, molecular profiles gathered from controlled conditions (e.g. greenhouse) capture the combined effect of the environment and the genotype, thus allowing for explaining a larger portion of a trait’s variance when they are used as predictors (the latter hinges on the assumption that the molecular traits can serve as determinants of the trait to be predicted). What our approach demonstrated is that metabolic (and possibly other) phenotypes in the two-step approach can result in hybrid prediction accuracies that exhibit smaller variability, likely attributed to the fact that these phenotypes manifest the joint effects of genotype and environment. However, due to availability of data from only two trails we did not attempt to determine the GxE component of HP and the extent to which it can be linked to metabolic profiles.

Altogether, our study demonstrates the great potential of young roots of maize as an adequate system for the metabolic prediction of HP in the field. The method is highly competitive compared to current breeding practices, saving time, space and costs in the testing routines.

## Materials and methods

### Plant material, metabolic and field data

The 381 genotypes under analysis derive from a partial factorial mating design with the European maize Dent × Flint heterotic pattern (24 Dents, 25 Flints and 332 hybrids from crosses thereof). The corresponding metabolic profiles from 3.5-day-old maize root samples, as well as whole-plant biomass (dt ha^-1^) from hybrids grown in two distinct field trials (2010 and 2012, BBCH stage 89) were previously reported [19]. To remove redundancy in the set of analytes, we identified all pairs of analytes with a Pearson correlation coefficient (*r*) greater than 0.85 and remove the analyte in each pair having the greatest average *r* with all other analytes.

### Encoding the metabolic inheritance pattern of analytes

Let *D* = {*d*_1_,*d*_2_,…,*d*_*n*_} and *F* = {*f*_1_,*f*_2_,…,*f*_*n*_} denote the sets of Dent and Flint inbred lines, respectively, and *H* = {(*d*_*i*_,*f*_*j*_) ∈ *D* × *F*} denote the incomplete set of the hybrids generated from crossing the *i*^th^ Dent inbred line with the *j*^th^ Flint inbred line.

For encoding the analytes levels in the hybrids, a *n* × *m* matrix carrying the metabolic profiles *X*_*PH*_ was first constructed, with *n* corresponding to the total number of replicates in all genotypes, and *m* the number of measured analytes. Then, |*H*| subsets of *X*_*PH*_ (each subset denoted by *X*_*s*_ ∈ {*X*_*s*,1_,*X*_*s*,2_,…,*X*_*s*,|*H*|_}) were generated, each carrying the replicates of the profiles of the Dent (*X*_*s*_(*d*,°)), the Flint (*X*_*s*_(*f*,°)) and the hybrid (*X*_*s*_(*h*,°)) from each individual cross. The analytes in each of these subsets were separately analyzed with the R package ‘limma’ [33] for significance and sign of the differences in the levels among Dent, Flint and hybrid, using moderate *t*-statistics without *P*-value adjustment. Finally, the *j*^th^ analyte in the *h*^th^ hybrid was encoded with a label *T*(*h*,*j*) ∈ {±2,±1,0} that denotes the metabolic inheritance pattern (mIP), using the following exhaustive conditionals,

- *X*_*s*_(*h*,*j*) > *X*_*s*_(*b*,*j*) AND *X*_*s*_(*h*,*j*) > *X*_*s*_(*w*,*j*) ⇒ *T*(*h*,*j*) = 2
- *X*_*s*_(*h*,*j*) = *X*_*s*_(*b*,*j*) AND *X*_*s*_(*h*,*j*) > *X*_*s*_(*w*,*j*) ⇒ *T*(*h*,*j*) = 1
- *X*_*s*_(*h*,*j*) = *X*_*s*_(*b*,*j*) AND *X*_*s*_(*h*,*j*) = *X*_*s*_(*w*,*j*) XOR
  *X*_*s*_(*h*,*j*) < *X*_*s*_(*b*,*j*) AND *X*_*s*_(*h*,*j*) > *X*_*s*_(*w*,*j*) ⇒ *T*(*h*,*j*) = 0
- *X*_*s*_(*h*,*j*) < *X*_*s*_(*b*,*j*) AND *X*_*s*_(*h*,*j*) = *X*_*s*_(*w*,*j*) ⇒ *T*(*h*,*j*) = −1
- *X*_*s*_(*h*,*j*) < *X*_*s*_(*b*,*j*) AND *X*_*s*_(*h*,*j*) < *X*_*s*_(*w*,*j*) ⇒ *T*(*h*,*j*) = −2

where *b* (resp. *w*) denote the parent in the set having the highest (resp. lowest) level relative to the other. Furthermore, ±2 corresponds to positive/negative overdominance, ±1 to positive/negative dominance and 0 to additivity, respectively. Disproportion in class frequencies was tackled by removing encoded analytes (i.e. every *T*(°,*j*)) having (*i*) any individual class represented in > 75% of the hybrids, and/or (*ii*) classes having less than four occurrences, unless a single class had a single occurrence, in which case we excluded the corresponding hybrid whenever classifying that particular analyte.

### Classification of the mIPs based on parental analytes

The predictor set consists of a matrix of size |*H*| × (2 × *m*), *X*_*DF*_, with *m* corresponding to the number of measured analytes, in which *X*_*DF*_(*h*,°) represents the concatenated metabolic profiles from the Flint and the Dent parents of the hybrid *h*. The levels were standardized column-wise.

In each separate encoded analyte *T*(°,*j*), the hybrid labels were predicted using seven different classifiers: linear discriminant analysis following a dimension-reducing partial least-squares transformation (PLS-DA) [22], logistic regression with the elastic net (glmnet) [23], random forests following a dimension-reducing partial least-squares transformation (PLS-RF) [24, 25], simple support vector machines with a radial basis function kernel (SVM) [26], support vector machines with a radial basis function kernel and class-specific weights (SVM-W), random forests (RF) [24] and random forests with class-specific weights (RF-W). Class-specific weights were symmetrically defined as *W* = {*w*_−*OD*_,*w*_−*D*_,*w*_*A*_,*w*_+*D*_,*w*_+*OD*_} = {10,5,1,5,10}. We repeated five times the following sequence: (*i*) create a stratified random partition of *X*_*DF*_ with 75% of the samples allocated to the training set and 25% to the test set, (*ii*) train the model with a 3-fold cross-validation (5 repetitions) to determine the tuning parameters that maximize Kappa [26] (S3 Table), (*iii*) use the optimal model to determine Kappa in the test set. The entire procedure was repeated with randomly permuted class labels in each of the encoded analytes.

The model exhibiting the lowest coefficient of variation of Kappa (CV = standard deviation / mean) over all encoded analytes was chosen for the subsequent metabolite ranking step. For this purpose, we trained the model with the entire predictor set (*X*_*DF*_) for predicting each *T*(°,*j*)separately, using a 3-fold cross-validation (10 repetitions) and extracted the relative variable importance (VIP). The parental analytes were then ranked by the corresponding median VIP across models for the different encoded analytes, scaled between 0 and 1. The entire procedure was conducted using the R package ‘caret’ [34].

### Prediction of field HP

Field hybrid performance (i.e. HP, whole-plant biomass) was predicted using support vector regression models (SVR, with a radial basis function kernel) [27], each with different subsets of parental features, using the previously computed ranking. For this purpose, we trained SVR with (*i*) the top 5 features, (*ii*) 5 randomly drawn from the top 10, (*iii*) 5 randomly drawn from the top 20, (*iv*) 5 randomly drawn from the top 50, (*v*) 5 randomly drawn from the top 100, (*vi*) 5 randomly drawn from the bottom 100, (*vii*) 5 randomly drawn from the bottom 50, (*viii*) 5 randomly drawn from the bottom 20, (*ix*) 5 randomly drawn from the bottom 10, (*x*) the bottom 5 features, (*xi*) 5 randomly drawn from the entire set, (*xii*) all features and finally (*xiii*) all features with permutation of the biomass data. We repeated 25 times the following sequence: (*i*) create a random partition of *X*_*DF*_ with 80% of the samples allocated to the training set and 20% to the test set, (*ii*) train the SVR with 3-fold cross-validation (10 repetitions) to determine the tuning parameters that maximize *R*^2^ (i.e. predictability, S4 Table), (*iii*) use the optimal model to determine *R*^2^ in the test set.

For classification, hybrids were split into ‘bad’ and ‘good’ performers, i.e. hybrids with biomass smaller and greater or equal to the average biomass value, respectively. The classification procedure was based on the sequence of steps described above, using support vector machines (SVM, with a radial basis function kernel) [26], aiming at maximizing accuracy and the area under the ROC (receiver operating characteristic) curves (AUC), separately. The AUC was calculated using the R package ‘pROC’ [35]. The precision-recall curves were computed for each of the different models, based on the class probabilities attributed to the hybrids in the corresponding test sets, using the R package ‘ROCR’ [36]. The entire modeling was conducted using the R package ‘caret’ [34].

### Code availability

Compiled R code is available from https://github.com/monogenea/HPmodelframework.

## Supporting information

S1 Fig Schematic representation of the classification-driven framework. (**a**) Selected Dent × Flint hybrid genotypes (D × F) and the corresponding Dent (D) and Flint (F) inbred parental lines were germinated under controlled conditions. (**b**) The primary roots from the germinated plants were subjected to gas chromatography separation followed by mass spectrometry (GC/MS) analysis. (**c**) For every available combination of D, D × F and F, the resulting metabolic profiles were compared to determine the metabolic inheritance patterns (mIPs) using the concepts of additivity (A), dominance (D) and overdominance (OD). (**d**) In each separate analyte, mIPs were predicted from the concatenated parental metabolic profiles. (**e**) Finally, parental analytes were ranked based on their importance in predicting mIPs. The top ranked analytes were selected for predicting hybrid performance in the field (HP). The data shown are purely illustrative. Icons are freely available at https://icons8.com/.
(PDF)

S2 Fig Accuracy in predicting ‘good’ and ‘bad’ performers based on subsets of the ranked parental features. Hybrid field performance was encoded into two groups (i.e. ‘bad’ and ‘good’ performers) and was predicted using support vector machines (SVM), each trained with different subsets of parental features, based on the median relative importance for predicting the heterotic mode of action. The models were trained with the top five parental features (‘Top 5’); five randomly drawn from the top 10/20/50/100 features (‘5 out of top 10’/‘5 out of top 20’/‘5 out of top 50’/‘5 out of top 100’, respectively); five randomly drawn from the bottom 10/20/50/100 features (‘5 out of bottom 10’/‘5 out of bottom 20’/‘5 out of bottom 50’/‘5 out of bottom 100’, respectively); bottom five parental features (‘Bottom 5’); five randomly drawn from all features (‘Random 5’). As the subsets comprise analytes ranked lower, the average test accuracy decreases. The full model (‘All’) exhibits a slightly smaller median accuracy compared to the top five parental features, whereas upon permutation of the values of hybrid performance (‘All (perm)’) the median accuracy is centered around 0.5.
(PDF)

S3 Fig Test of robustness of different cutoff values for binning ‘bad’ and ‘good’ hybrids. Nine evenly-spaced threshold values of hybrid performance (HP), including the mean, were used to bin ‘bad’ and ‘good’ hybrids used for classification of HP. Accuracy (top) decreases as values spread away from the average value (i.e. 592 dt ha^-1^) and increases again with more extreme values. This is an artifact considered by the Kappa metric (bottom), which shows the greatest accuracy is attained with the mean HP value.
(PDF)

S4 Fig Area under the curve (AUC) in predicting ‘good’ and ‘bad’ performers based on subsets of the ranked parental features. Hybrid field performance was encoded into two groups (i.e. ‘bad’ and ‘good’ performers) and predicted using support vector machines (SVM), each trained with different subsets of parental features, based on the median relative importance for predicting the heterotic mode of action. The models were trained with the top five parental features (‘Top 5’); five randomly drawn from the top 10/20/50/100 features (‘5 out of top 10’/‘5 out of top 20’/‘5 out of top 50’/‘5 out of top 100’, respectively); five randomly drawn from the bottom 10/20/50/100 features (‘5 out of bottom 10’/‘5 out of bottom 20’/‘5 out of bottom 50’/‘5 out of bottom 100’, respectively); bottom five parental features (‘Bottom 5’); five randomly drawn from all features (‘Random 5’). As the subsets comprise analytes ranked lower, the average test AUC decreases. The full model (‘All’) exhibits a slightly higher average AUC compared to the top five parental features, whereas upon permutation of the values of hybrid performance (‘All (perm)’) the average AUC is substantially lower.
(PDF)

S5 Fig Cumulative relative frequencies of the Dent (maternal) and Flint (parental) analytes along the ranking. The cumulative relative frequency of the Dent (maternal, red) analytes is systematically higher than that of the Flint (paternal, grey) analytes along the ranking (i.e. increasing values in the *x*-axis).
(PDF)

S6 Fig Effect of miP class balance threshold relaxation on the predictability of hybrid performance based on subsets of the ranked parental features. Hybrid field performance was predicted using support vector regression models (SVR), each trained with different subsets of parental features, based on the median relative importance for predicting the metabolic inheritance patterns (mIPs) that pass a class balance threshold of 0.9, as opposed to 0.75. The models were trained with the top five parental features (‘Top 5’); five randomly drawn from the top 10/20/50/100 features (‘5 out of top 10’/‘5 out of top 20’/‘5 out of top 50’/‘5 out of top 100’, respectively); five randomly drawn from the bottom 10/20/50/100 features (‘5 out of bottom 10’/‘5 out of bottom 20’/‘5 out of bottom 50’/‘5 out of bottom 100’, respectively); bottom five parental features (‘Bottom 5’); five randomly drawn from all features (‘Random 5’). As the subsets comprise analytes ranked lower, predictability (*R*^2^) decreases. The full model (‘All’) exhibits the highest median *R*^2^ whereas upon permutation of the values of hybrid performance (‘All (perm)’) the median *R*^2^ is almost null.
(PDF)

S1 Dataset Metabolic (log10-transformed metabolite intensities) and biomass data (dt ha^-^1) with field trial designation. (XLSX)

S1 Table Encoded analytes and distribution of mode of inheritance classes. Rows correspond to the 136 analytes, whereas columns correspond to the 332 crosses. Red, orange, yellow, blue and navy blue colors represent positive overdominance, positive dominance, additivity, negative dominance and negative overdominance, respectively.
(XLSX)

S2 Table Coefficient of variation (CV) and average Kappa per model and encoded analyte. Coefficient of variation (CV = standard deviation / mean) and average Kappa across the five independent test repetitions. The 41 encoded analytes are sorted by decreasing order of the average Kappa (AVE KAPPA).
(XLSX)

S3 Table Model parameterization for classification of mIPs. The table summarizes the parameters tuned for maximizing the cross-validated Kappa with each of the seven methods. Except PLS-DA, all methods were tuned using two parameters each.
(XLSX)

S4 Table Model parameterization for prediction of hybrid performance. The table summarizes the parameters tuned for maximizing the cross-validated R2 (resp. accuracy, AUC) with SVR (resp. SVM).
(XLSX)

S1 Note (DOCX)
