# Supplementary material for: Classification-driven framework to predict maize hybrid field performance from metabolic profiles of young parental roots
Source: PLoS One. 2018 Apr 26;13(4):e0196038. doi: 10.1371/journal.pone.0196038 (PMC5919381; doi:10.1371/journal.pone.0196038)
Supplement: S1 Fig — (a) Selected Dent × Flint hybrid genotypes (D × F) and the corresponding Dent (D) and Flint (F) inbred parental lines were germinated under controlled conditions. (b) The primary roots from the germinated plants were subjected to gas chromatography separation followed by mass spectrometry (GC/MS) analysis. (c) For every available combination of D, D × F and F, the resulting metabolic profiles were compared to determine the metabolic inheritance patterns (mIPs) using the concepts of additivity (A), dominance (D) and overdominance (OD). (d) In each separate analyte, mIPs were predicted from the concatenated parental metabolic profiles. (e) Finally, parental analytes were ranked based on their importance in predicting mIPs. The top ranked analytes were selected for predicting hybrid performance in the field (HP). The data shown are purely illustrative. Icons are freely available at https://icons8.com/. (PDF) [file pone.0196038.s001.pdf]

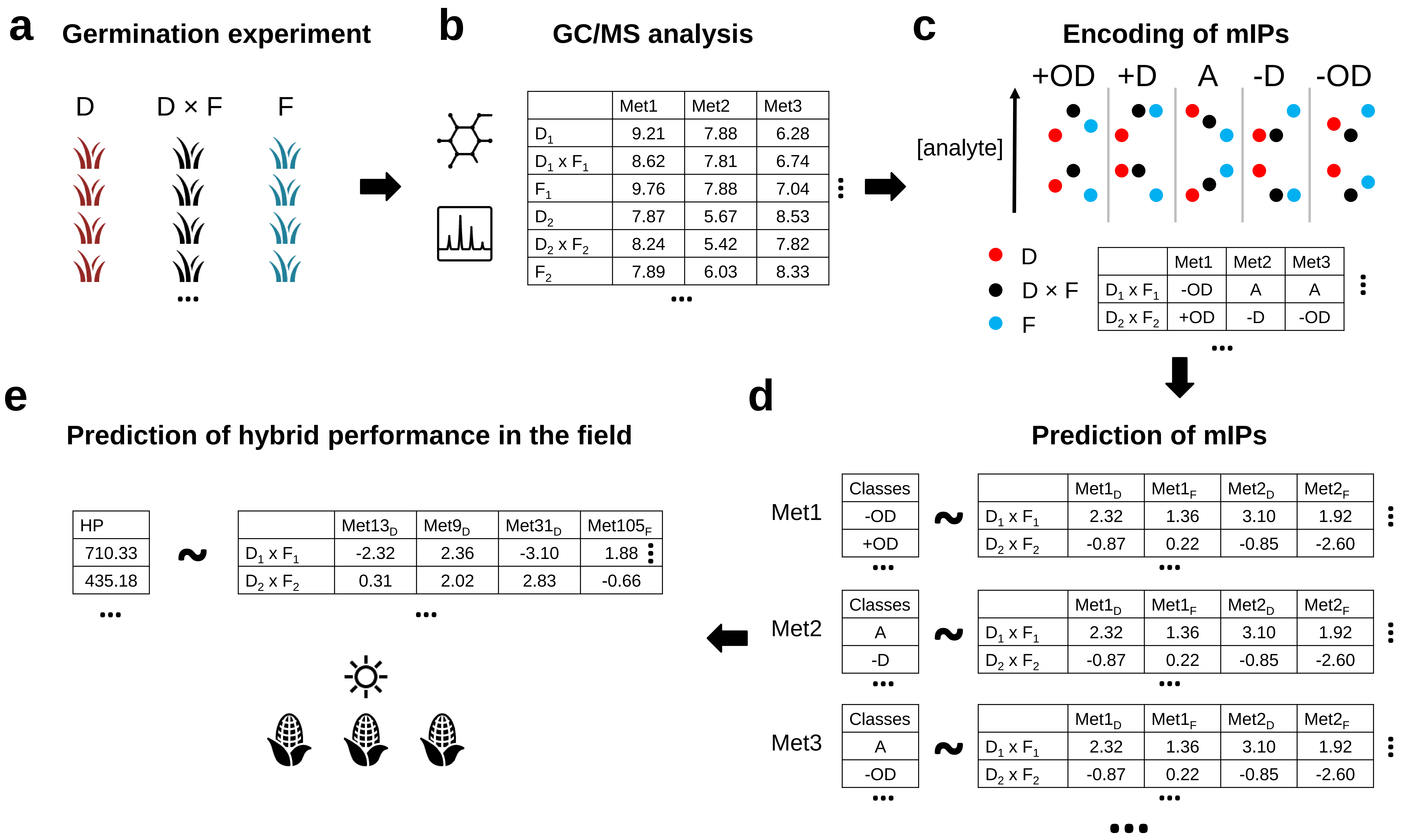
a
Germination experiment
D
D × F
F
b
GC/MS analysis
Met1 Met2 Met3
D1 9.21 7.88 6.28
D1 x F1 8.62 7.81 6.74
F1 9.76 7.88 7.04
D2 7.87 5.67 8.53
D2 x F2 8.24 5.42 7.82
F2 7.89 6.03 8.33
c
Encoding of mIPs
+OD +D A -D -OD
[analyte]
D
D × F
F
Met1 Met2 Met3
D1 x F1 -OD A A
D2 x F2 +OD -D -OD
d
Prediction of mIPs
Met1
Classes
-OD
+OD
Met1D Met1F Met2D Met2F
D1 x F1 2.32 1.36 3.10 1.92
D2 x F2 -0.87 0.22 -0.85 -2.60
Met2
Classes
A
-D
Met1D Met1F Met2D Met2F
D1 x F1 2.32 1.36 3.10 1.92
D2 x F2 -0.87 0.22 -0.85 -2.60
Met3
Classes
A
-OD
Met1D Met1F Met2D Met2F
D1 x F1 2.32 1.36 3.10 1.92
D2 x F2 -0.87 0.22 -0.85 -2.60
e
Prediction of hybrid performance in the field
HP
710.33
435.18
Met13D Met9D Met31D Met105F
D1 x F1 -2.32 2.36 -3.10 1.88
D2 x F2 0.31 2.02 2.83 -0.66
