# Supplementary figures and images for: Classification-driven framework to predict maize hybrid field performance from metabolic profiles of young parental roots
Source: PLoS One. 2018 Apr 26;13(4):e0196038. doi: 10.1371/journal.pone.0196038 (PMC5919381; doi:10.1371/journal.pone.0196038)

Accuracy

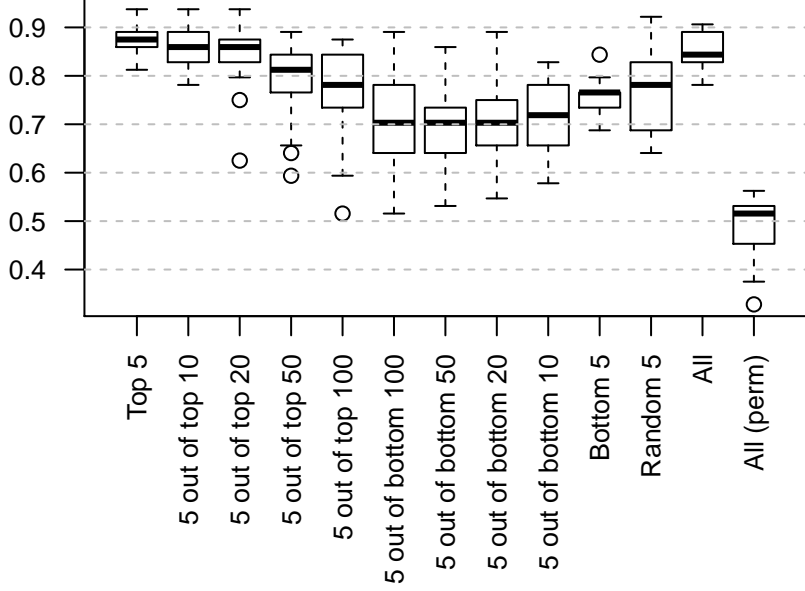

Supplement: S2 Fig — Hybrid field performance was encoded into two groups (i.e. ‘bad’ and ‘good’ performers) and was predicted using support vector machines (SVM), each trained with different subsets of parental features, based on the median relative importance for predicting the heterotic mode of action. The models were trained with the top five parental features (‘Top 5’); five randomly drawn from the top 10/20/50/100 features (‘5 out of top 10’/‘5 out of top 20’/‘5 out of top 50’/‘5 out of top 100’, respectively); five randomly drawn from the bottom 10/20/50/100 features (‘5 out of bottom 10’/‘5 out of bottom 20’/‘5 out of bottom 50’/‘5 out of bottom 100’, respectively); bottom five parental features (‘Bottom 5’); five randomly drawn from all features (‘Random 5’). As the subsets comprise analytes ranked lower, the average test accuracy decreases. The full model (‘All’) exhibits a slightly smaller median accuracy compared to the top five parental features, whereas upon permutation of the values of hybrid performance (‘All (perm)’) the median accuracy is centered around 0.5. (PDF) [file pone.0196038.s002.pdf]

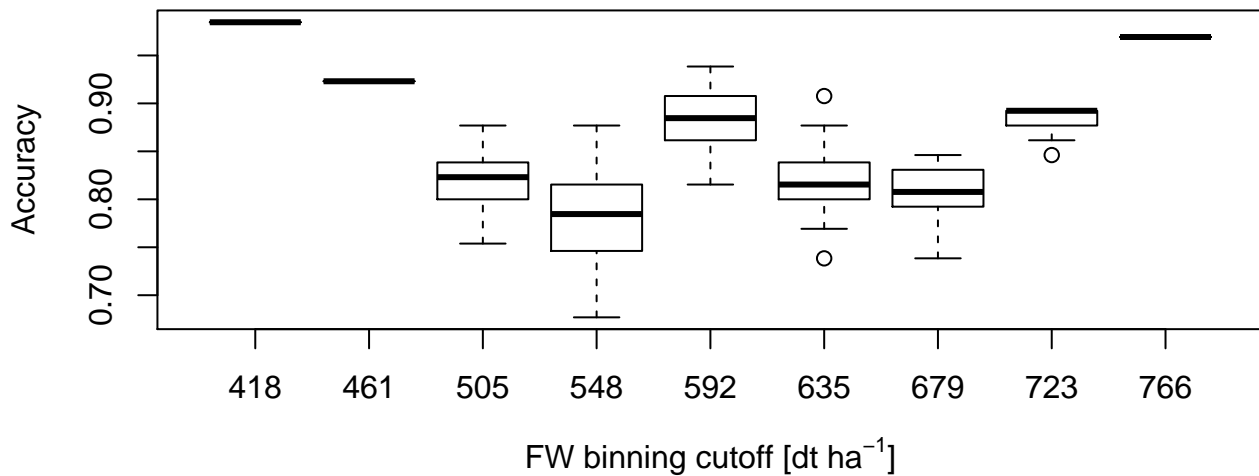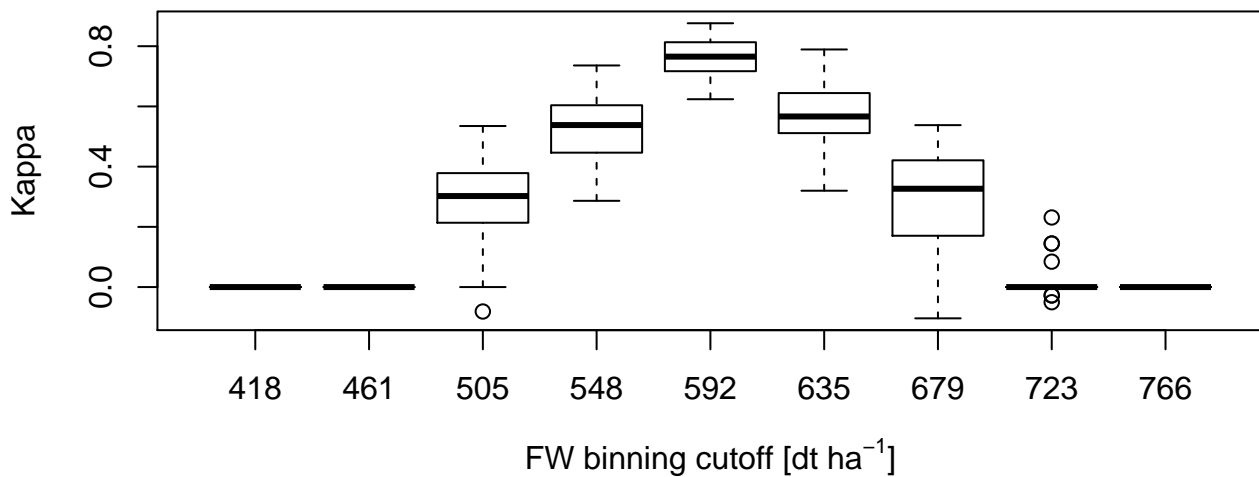

Supplement: S3 Fig — Nine evenly-spaced threshold values of hybrid performance (HP), including the mean, were used to bin ‘bad’ and ‘good’ hybrids used for classification of HP. Accuracy (top) decreases as values spread away from the average value (i.e. 592 dt ha-1) and increases again with more extreme values. This is an artifact considered by the Kappa metric (bottom), which shows the greatest accuracy is attained with the mean HP value. (PDF) [file pone.0196038.s003.pdf]

Area under the ROC curve (AUC)

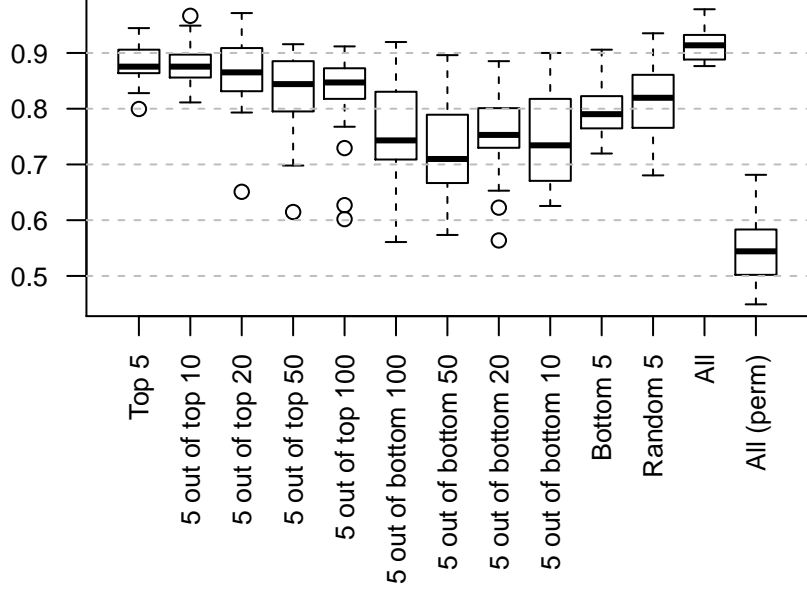

Supplement: S4 Fig — Hybrid field performance was encoded into two groups (i.e. ‘bad’ and ‘good’ performers) and predicted using support vector machines (SVM), each trained with different subsets of parental features, based on the median relative importance for predicting the heterotic mode of action. The models were trained with the top five parental features (‘Top 5’); five randomly drawn from the top 10/20/50/100 features (‘5 out of top 10’/‘5 out of top 20’/‘5 out of top 50’/‘5 out of top 100’, respectively); five randomly drawn from the bottom 10/20/50/100 features (‘5 out of bottom 10’/‘5 out of bottom 20’/‘5 out of bottom 50’/‘5 out of bottom 100’, respectively); bottom five parental features (‘Bottom 5’); five randomly drawn from all features (‘Random 5’). As the subsets comprise analytes ranked lower, the average test AUC decreases. The full model (‘All’) exhibits a slightly higher average AUC compared to the top five parental features, whereas upon permutation of the values of hybrid performance (‘All (perm)’) the average AUC is substantially lower. (PDF) [file pone.0196038.s004.pdf]

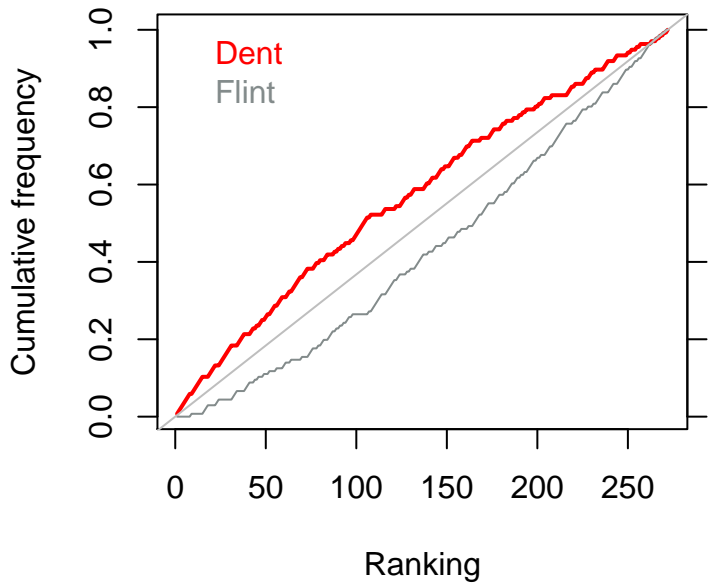

Supplement: S5 Fig — The cumulative relative frequency of the Dent (maternal, red) analytes is systematically higher than that of the Flint (paternal, grey) analytes along the ranking (i.e. increasing values in the x-axis). (PDF) [file pone.0196038.s005.pdf]

$R^2$ 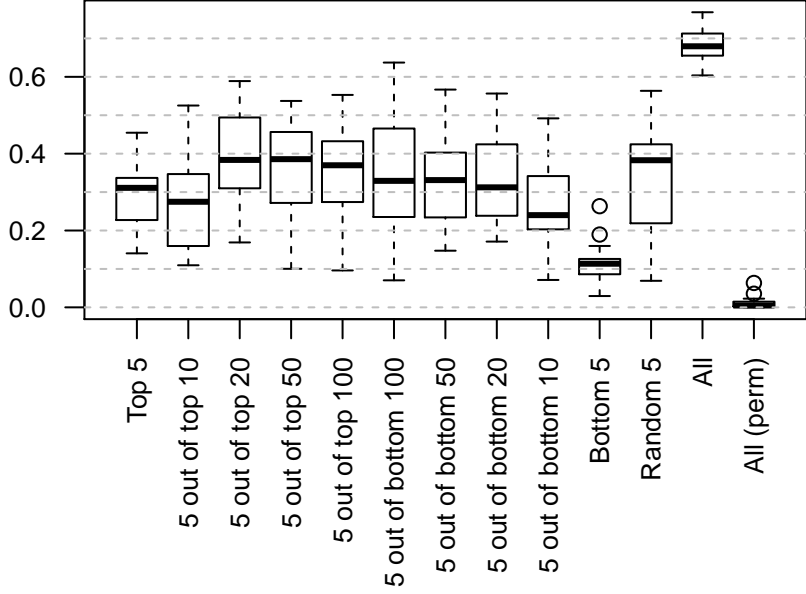

Supplement: S6 Fig — Hybrid field performance was predicted using support vector regression models (SVR), each trained with different subsets of parental features, based on the median relative importance for predicting the metabolic inheritance patterns (mIPs) that pass a class balance threshold of 0.9, as opposed to 0.75. The models were trained with the top five parental features (‘Top 5’); five randomly drawn from the top 10/20/50/100 features (‘5 out of top 10’/‘5 out of top 20’/‘5 out of top 50’/‘5 out of top 100’, respectively); five randomly drawn from the bottom 10/20/50/100 features (‘5 out of bottom 10’/‘5 out of bottom 20’/‘5 out of bottom 50’/‘5 out of bottom 100’, respectively); bottom five parental features (‘Bottom 5’); five randomly drawn from all features (‘Random 5’). As the subsets comprise analytes ranked lower, predictability (R2) decreases. The full model (‘All’) exhibits the highest median R2 whereas upon permutation of the values of hybrid performance (‘All (perm)’) the median R2 is almost null. (PDF) [file pone.0196038.s006.pdf]
