# Supplementary material for: Classification-driven framework to predict maize hybrid field performance from metabolic profiles of young parental roots
Source: PLoS One. 2018 Apr 26;13(4):e0196038. doi: 10.1371/journal.pone.0196038 (PMC5919381; doi:10.1371/journal.pone.0196038)
Supplement: S1 Note — (DOCX) [file pone.0196038.s012.docx]

**S1 Note**

It has been reported that nonadditive patterns may be associated with hybrid performance [1]. To test for such association in our experimental design, we examined the relationship of hybrid performance with the relative frequency of nonadditive labels (i.e. dominance and overdominance) in the selected analytes. We observed no significant correlation between the two (*r* = 0.032, two-tailed *t*-test *P* = 0.56), suggesting that global metabolic nonadditive inheritance cannot explain the variation in hybrid performance (Fig 1).


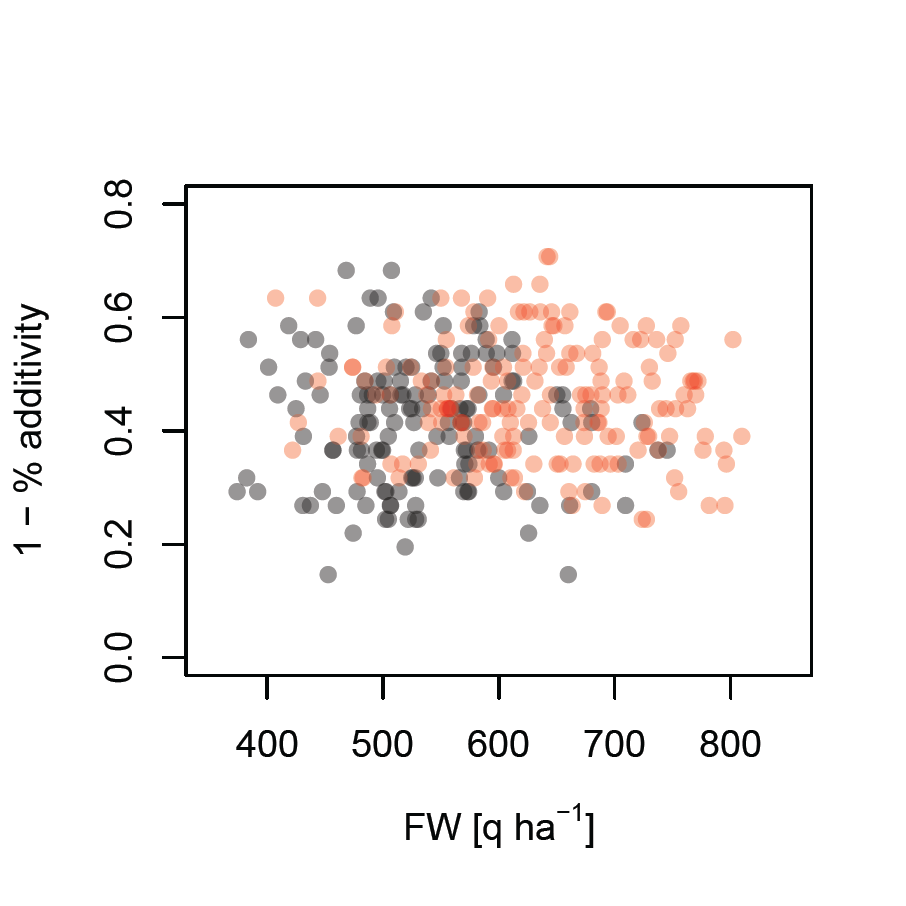


**Fig 1. Relationship between hybrid performance and metabolic nonadditive inheritance.** The sum of the relative frequencies of nonadditive classes (*i.e.* $\pm$ dominance and $\pm$ overdominance) in hybrids was compared to the respective field performance. No significant correlation was observed (*r* = 0.032, two-tailed *t*-test *P* = 0.56). Grey and red characters represent hybrids evaluated in the trials of 2010 and 2012, respectively.

1. Seymour DK, Chae E, Grimm DG, Martín Pizarro C, Habring-Müller A, Vasseur F, Rakitsch B, Borgwardt KM, Koenig D, Weigel D (2016) Genetic architecture of nonadditive inheritance in Arabidopsis thaliana hybrids. *Proceedings of the National Academy of Sciences* **113:** E7317-E7326
